# Chronotype delay and sleep disturbances shaped by the Antarctic polar night

**DOI:** 10.1038/s41598-023-43102-0

**Published:** 2023-09-24

**Authors:** C. Tortello, A. Folgueira, J. M. Lopez, F. Didier Garnham, E. Sala Lozano, M. S. Rivero, G. Simonelli, D. E. Vigo, S. A. Plano

**Affiliations:** 1 Chronophysiology Lab, Institute for Biomedical Research (BIOMED), Pontifical Catholic University of Argentina (UCA), National Scientific and Technical Research Council (CONICET), Buenos Aires, Argentina; 2 Health Department of Armed Force Personnel, Ministry of Defense, Buenos Aires, Argentina; 3 Argentine Joint Command, Buenos Aires, Argentina; 4 Argentine Antarctic Institute, Buenos Aires, Argentina; 5 Département de Médecine, Faculté de Médecine, Université de Montréal, Montreal, Canada; 6 Département de Neurosciences, Faculté de Médecine, Université de Montréal, Montreal, Canada; 7 Centre d’études Avancées en Médecine du Sommeil, Hôpital du Sacré-Coeur de Montréal, CIUSSS NÎM, Montreal, Canada; 8 Faculty of Psychology and Educational Sciences, Katholieke Universiteit Leuven, Leuven, Belgium; 9 Chronobiology Lab, Department of Science and Technology, National University of Quilmes (UNQ), Buenos Aires, Argentina

**Keywords:** Circadian regulation, Sleep

## Abstract

Chronotype is a reliable biomarker for studying the influence of external zeitgebers on circadian entrainment. Assessment of chronotype variation in participants exposed to extreme photoperiods may be useful to investigate how changes in light–dark cycle modulate the circadian system. This study aimed to examine chronotype and sleep changes during a winter campaign at the Argentine Antarctic station Belgrano II. A sample of 82 men who overwintered in Antarctica completed the Munich Chronotype Questionnaire during March (daylight length: 18.6 h), May (daylight length: 2.8 h), July (daylight length: 0 h), September (daylight length: 14.5 h), November (daylight length: 24 h). The main results showed a decrease in sleep duration and a delay in chronotype and social jetlag during the polar night, highlighting the influence of social cues and the impact of the lack of natural light on circadian rhythms.

## Introduction

Circadian rhythms are driven by a complex network of independent oscillators coordinated with each other by the suprachiasmatic nucleus and entrained by external zeitgebers^1,2^. Therefore, assessing a single individual´s phase of entrainment represents a challenging task to achieve. However, the timing of biological processes such as acrophase, dim-light melatonin onset, or chronotype^3^, can serve as biomarkers to estimate the phase of this complex and multi-oscillating system.

Chronotype, as a biological construct partially determined by genetics, is defined as a phenotype that reflects human preferences in the timing of sleep and wake^4^. The Morningness-Eveningness Questionnaire (MEQ), developed by Horne and Östberg^5^, stands as the first validated instrument for assessing chronotype. This questionnaire measures an individual’s daily behavioral preference on an ordinal scale, grounded in the idea that chronotype embodies a personality trait^6^. Several other procedures to measure chronotype have been developed, though the Munich ChronoType Questionnaire (MCTQ) has proved to be one of the most reliable. Designed by Roenneberg et al. (2003) the MCTQ measures the chronotype according to the reported middle point of the sleep period (mid-sleep) of the work-free days^7^. This questionnaire correlates well with data taken from sleep logs^8^, wrist actimetry^9^, and dim-light melatonin onset (the gold standard for phase determination, but also an expensive and laborious technique to implement)^10^. Furthermore, the advantage of being a self-administrated questionnaire enhances the possibility of assessing it in a more significant number of samples. This robust and versatile instrument showed to be useful for studying the epidemiology of the human circadian clock^11^, measuring chronotype repeatedly over time^12^, detecting changes related to geographical location^13^, predicting school performances in adolescents^14^, and evaluating cardiometabolic disease in night workers^15^.

Light exposure is considered to be the main zeitgeber that synchronizes the central clock. In order to assess the impact of the light–dark cycle on the circadian system, several studies have focused on the influence of the photoperiod on chronotype variations^16,17^. Differences in chronotypes by latitudes have been described, showing that the timing of this biomarker is at least, partially, dependent on this environmental factor^18,19^. Extreme photoperiods where either day or night tend to last longer or even not exist at all (i.e. the sun does not rise above the horizon during the polar night or the sun does not descend below the horizon during the polar day) have shown to modulate this phase of entrainment. For example, in a sub-Arctic region, chronotype evidenced a delay during dark periods^20^.

Antarctica has gained interest in chronobiological research as it offers an ideal setting to ecologically study circadian parameters in extreme light–dark cycle conditions. Physiological and behavioral changes have been reported because of Antarctic isolation and extreme photoperiods^21^. The study of the impact of polar night on chronotypes has shown mixed results. Some studies have shown a delay in mid-sleep^22^, while others have revealed that most participants defined themselves as “definitely morning” chronotype during this period^23^. Differences in chronotypes have also been studied during summer campaigns, showing a significant decrease in mid-sleep in Antarctica compared to measures taken before and after traveling to the `white continent´ as well as greater social jetlag for late chronotypes^24^. Also, variations in dim-light melatonin onset have been found between chronotypes, with a tendency for early chronotypes to be delayed in comparison with late ones, which showed an advanced phase^25^.

As exposed above, few studies have focused on chronotypes during Antarctic expeditions and, those that have assessed them, generally included small samples, self-perceived preferences, short periods of exposure, or Antarctic locations with milder photoperiods. On the other hand, previous studies have shown sleep changes caused mainly by the absence of natural light exposure during Antarctic polar night, evidenced in shorter sleep duration due to delayed bedtime^26^. Although these sleep variations can be modulated by chronotype and seasonality^27^, no studies have previously explored this association under extreme photoperiods during a winter campaign in Antarctica.

Therefore, our primary objective was to longitudinally assess chronotype (MSFsc) variations according to the MCTQ during the months of March (daylight length: 18.6 h), May (daylight length: 2.8 h), July (daylight length: 0 h), September (daylight length: 14.5 h), and November (daylight length: 24 h) at Belgrano II Argentine Antarctic station, utilizing data from a pooled sample across multiple campaigns. As secondary objectives, we assessed the influence of the day length and the chronotype variations on sleep variables. The main hypothesis was that participants show a significant delay in chronotype and an increase in social jetlag during the polar night due to the lack of natural light.

## Results

Descriptive analyses of the chronotype and sleep measures are presented in Table 1.

**Table 1:** Mean and standard deviation of chronotype and sleep variables throughout the Antarctic campaigns.

		March	May	July	September	November
		Mean (SE)	Mean (SE)	Mean (SE)	Mean (SE)	Mean (SE)
MSFsc [Clk]		4.8 (0.1)	5.1 (0.1)	5.2 (0.1)	4.9 (0.1)	5 (0.1)
Mid-sleep [Clk]	(W)	4 (0.1)	4.1 (0.1)	4.1 (0.1)	4.1 (0.1)	4.1 (0.1)
	(F)	5 (0.1)	5.3 (0.1)	5.5 (0.1)	5.1 (0.1)	5.2 (0.1)
Social Jetlag [m]		62.2 (6.3)	73.4 (6.5)	82.1 (7.5)	61.7 (6.1)	65.8 (5.9)
Sleep duration [m]	(W)	437.2 (8)	423.4 (7.4)	424.4 (7.3)	431.3 (8.2)	435.7 (8.3)
	(F)	455.2 (10.6)	422.7 (8.4)	444.8 (9.3)	444.8 (11.7)	440.8 (8.6)
Nap [m]	(W)	47.3 (3.5)	61.9 (5.5)	61.3 (6.4)	59.7 (6.4)	45.2 (4.7)
	(F)	64.5 (5.4)	62.4 (5.9)	70.8 (6.6)	63.7 (6.2)	56.1 (5.4)
Total sleep [m]	(W)	437.2 (8.7)	423.4 (9)	424.4 (8.5)	431.3 (7.6)	435.7 (8.9)
	(F)	455.2 (11.4)	422.7 (9.6)	444.8 (10.9)	444.8 (11.3)	440.8 (9.4)
Sleep onset [Clk]	(W)	0.3 (0.1)	0.6 (0.1)	0.6 (0.1)	0.6 (0.1)	0.4 (0.1)
	(F)	1.3 (0.2)	1.8 (0.1)	1.8 (0.1)	1.5 (0.2)	1.5 (0.1)
Sleep offset [Clk]	(W)	7.6 (0.1)	7.6 (0.1)	7.6 (0.1)	7.7 (0.1)	7.7 (0.1)
	(F)	8.8 (0.2)	8.9 (0.1)	9.3 (0.2)	8.9 (0.1)	8.9 (0.1)
Sleep onset latency [m]	(W)	13.6 (1.1)	14.2 (1.1)	15.6 (1.1)	15 (1.1)	13.9 (1.1)
	(F)	14.1 (1.2)	14.2 (1.1)	14.5 (1.1)	14.6 (1)	13.9 (1.1)
Sleep inertia [Clk]	(W)	7.9 (0.1)	7.9 (0.1)	8 (0.1)	8 (0.1)	7.8 (0.2)
	(F)	9.2 (0.2)	9.6 (0.2)	9.4 (0.3)	9.5 (0.2)	8.9 (0.3)
Time to bed [Clk]	(W)	0.1 (0.1)	0.3 (0.1)	0.3 (0.1)	0.3 (0.1)	0.2 (0.1)
	(F)	1.1 (0.2)	1.6 (0.1)	1.6 (0.1)	1.3 (0.2)	1.3 (0.1)

*[m] = minutes; [Clk] = clock time.

**Figure 1:**
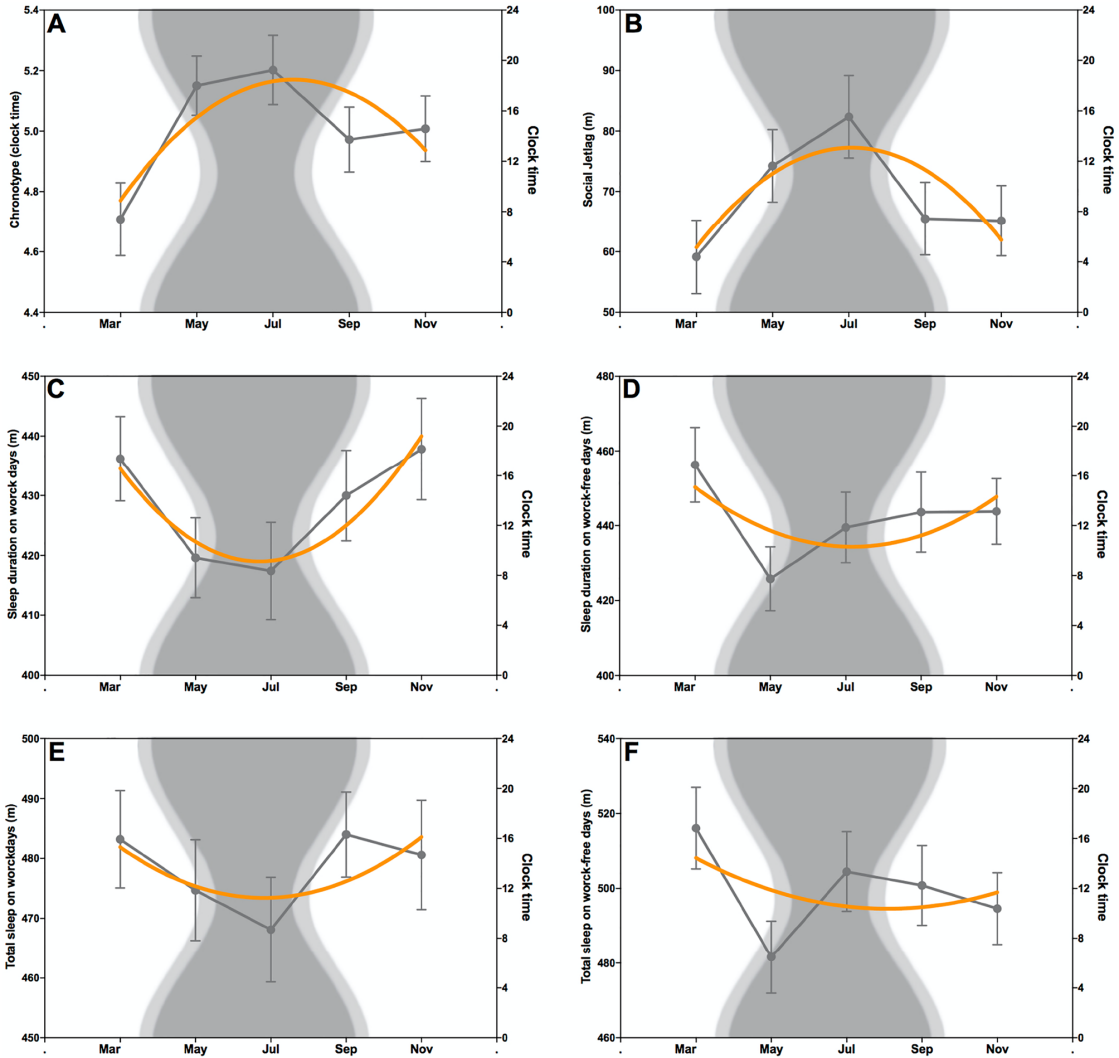
Circadian and sleep variations in Belgrano II. The curves in orange show the predicted values for Chronotype (**A**), Social Jetlag (**B**), Sleep duration on workdays (**C**), Sleep duration on work-free days (**D**), Total sleep on workdays (**E**), Total sleep on work-free days (**F**) during each measurement point. The grey lines show the mean and SEM of the respective variables. In the background, the photoperiod throughout the year is represented using three different colors: daylight is depicted in white, civil twilight in light grey and night in dark grey (https://www.timeanddate.com). The respective times throughout the day are shown on the right Y-axis, represented in clock time.

Results for the main effects of Model 1, which focus on a seasonal modulation of the circadian and sleep variables derived from a winter campaign are presented in Table 2. MSFsc showed a quadratic growth throughout the year with greater values during polar night, exhibiting the influence of the duration of natural light hours on the circadian system, as evidenced by the delay of this phase of entrainment (*p* < 0.001) (Fig. 1A). Social Jetlag also increased during shortened day length revealing the impact of the lack of a clear light clue in the differences between work and work-free days (*p* < 0.001) (Fig. 1B). In contrast, a U-shape pattern was observed for Sleep duration on workdays also evidencing a quadratic growth in which minimum sleep duration values were observed during winter (*p* < 0.001) (Fig. 1C). Remarkably, this significant effect for night sleep disappeared when the nap duration was included, as shown in Total Sleep on workdays (*p* = ns) (Fig. 1E). Neither Sleep duration nor Total Sleep on work-free days showed significant variability considering day length fluctuations (*p* = ns; *p* = ns) (Fig. 1D, F). Additionally, Sleep onset and Time to bed on workdays exhibited significantly later values associated with shorter day length (*p* = 0.01; *p* < 0.01, respectively) while during work-free days the pattern was even more pronounced (*p* < 0.001; *p* < 0.001). Similarly, Sleep offset on work-free days shifted to later hours in response to decreased daylight (*p* < 0.01). Neither Sleep onset latency nor Sleep inertia showed significant variations throughout the campaign (*p* = ns; *p* = ns; respectively).

**Table 2:** Parameters estimates for HLM models of chronotype and sleep variables.

			Model 1			Model 2			
			Intercept	Month	Day length	Intercept	Month	Day length	Chronotype
MSFsc		Coefficient (SE)	4.9 (0.1)	0.1 (0.02)	− 0.02 (0.003)				
		*p*	< 0.001	< 0.01	< 0.001				
Mid-sleep	W	Coefficient (SE)	243.1 (4)	1.6 (1)	− 0.2 (0.1)				
		*p*	< 0.001	0.1	0.1				
	F	Coefficient (SE)	314.2 (7)	4.4 (1)	− 1.2 (0.2)				
		*p*	< 0.001	< 0.01	< 0.001				
Social Jetlag		Coefficient (SE)	71.3 (6)	2.8 (1)	− 1 (0.2)	− 150.8 (10)	− 1 (1)	− 0.1 (0.1)	44.7 (2)
		*p*	< 0.001	0.06	< 0.001	< 0.001	0.3	0.3	< 0.001
Sleep duration	W	Coefficient (SE)	419.1 (8)	− 1.2 (2)	1 (0.2)	422.5 (19)	− 0.6 (2)	1 (0.2)	− 1.4 (3)
		*p*	< 0.001	0.5	< 0.001	< 0.001	0.7	< 0.001	0.7
	F	Coefficient (SE)	435 (10)	− 1.7 (2)	1 (0.2)	507 (25)	− 0.4 (2)	0.5 (0.3)	− 14.5 (5)
		*p*	< 0.001	0.5	0.02	< 0.001	0.8	0.1	< 0.01
Total sleep	W	Coefficient (SE)	480.1 (9)	− 1.4 (2)	0.4 (0.3)	470.8 (22)	− 1.5 (2)	0.6 (0.3)	1.3 (4)
		*p*	< 0.001	0.5	0.2	< 0.001	0.5	0.1	0.7
	F	Coefficient (SE)	505.1 (12)	− 2.1 (3)	0.4 (0.5)	553.1 (30)	− 1.4 (3)	.2 (0.5)	− 9.6 (6)
		*p*	< 0.001	0.5	0.4	< 0.001	0.6	0.6	0.1
Sleep onset	W	Coefficient (SE)	1468 (7)	2.2 (2)	− 1 (0.2)	1401.7 (16)	0.4 (1)	− 0.2 (0.2)	13.3 (3)
		*p*	< 0.001	0.2	0.01	< 0.001	0.8	0.3	< 0.001
	F	Coefficient (SE)	1532.6 (8)	5.4 (2)	− 1.5 (0.3)	1210.3 (11)	− 0.2 (1)	− 0.3 (1)	64.9 (2)
		*p*	< 0.001	< 0.01	< 0.001	< 0.001	0.8	0.05	< 0.001
Sleep offset	W	Coefficient (SE)	457.1 (7)	2.5 (2)	− 0.2 (0.3)	395.2 (10)	0.2 (1)	0.5 (0.1)	11.2 (2)
		*p*	< 0.001	0.2	0.4	< 0.001	0.7	< 0.001	< 0.001
	F	Coefficient (SE)	535 (9)	3.8 (2)	− 1 (0.3)	282.8 (16)	− 0.6 (2)	0.1 (0.2)	50.1 (3)
		*p*	< 0.001	0.1	< 0.01	< 0.001	0.7	0.6	< 0.001
Sleep onset latency	W	Coefficient (SE)	15.6 (1)	− 0.04 (0.3)	− 0.05 (0.05)	9.3 (3)	− 0.1 (0.3)	− 0.02 (0.05)	1.2 (0.6)
		*p*	< 0.001	0.9	0.4	< 0.01	0.8	0.6	< 0.05
	F	Coefficient (SE)	15.2 (1)	0.01 (0.2)	0.04 (0.04)	13.1 (3)	− 0.03 (0.2)	− 0.04 (0.04)	0.4 (0.5)
		*p*	< 0.001	0.9	0.2	< 0.001	0.9	0.3	0.4
Sleep inertia	W	Coefficient (SE)	480.2 (9)	1.7 (3)	− 0.6 (0.4)	403.8 (19)	− 1 (2)	0.2 (0.3)	14.2 (4)
		*p*	< 0.001	0.5	0.2	< 0.001	0.7	0.5	< 0.001
	F	Coefficient (SE)	560 (15)	− 1.9 (4)	− 0.3 (0.6)	384.2 (35)	− 4.2 (4)	0.2 (0.6)	35.7 (7)
		*p*	< 0.001	0.6	0.7	< 0.001	0.2	0.7	< 0.001
Time to bed	W	Coefficient (SE)	1454.8 (7)	2.2 (2)	− 0.1 (0.2)	1390.7 (17)	1 (2)	− 0.3 (0.2)	12.7 (3)
		*p*	< 0.001	0.2	0.01	< 0.001	0.7	0.2	< 0.001
	F	Coefficient (SE)	1518.4 (8)	5.5 (2)	− 1.5 (0.3)	1199.3 (11)	− 0.1 (1)	− 0.4 (0.2)	64.3 (2)
		*p*	< 0.001	< 0.01	< 0.001	< 0.001	0.9	< 0.05	< 0.001

*SE = Standard Error, W = Workdays, F = Work-free days.

To elucidate the effect of the chronotype on sleep variables, we included it as a level 1 predictor (Table 2). We observed that MSFsc evidenced a significant main effect on Antarctic participants’ Social Jetlag and Sleep duration on work-free days. Regarding the relationship between MSFsc and Social Jetlag, the model showed a significant modulation of participants` social jetlag values, showing that MSFsc increased for each unit increase in Social Jetlag (*p* < 0.001). In contrast, Sleep duration on work-free days evidenced a reduction of chronotype values for each unit increase in sleep duration during work-free days (*p* < 0.01). In summary, later chronotypes showed greater social jetlag and longer periods of sleep during work-free days. Sleep onset, Time to bed and Sleep offset exhibited consistent patterns, indicating that individuals with later chronotypes experienced later sleep phases on both workdays (*p* < 0.001; *p* < 0.001, respectively) and work-free days (*p* < 0.001; *p* < 0.001, respectively). MSFsc also revealed a significant effect on Sleep inertia on workdays and work-free days, evidencing a delayed in these phases for each unit of increase in chronotype values (*p* < 0.001; *p* < 0.001, respectively). In the same line, Sleep onset latency on workdays showed prolonged durations for later chronotypes (*p* < 0.05).

## Discussion

This is the first study to longitudinally analyze chronotype and sleep variables with the MCTQ during a winter campaign in Antarctica, using a large sample size. The main findings of the research were that Chronotype, Social Jetlag, and Sleep duration during workdays showed a significant seasonal variation modulated by the length of the day.

### Model 1. Seasonal modulation

Results from Model 1 supported our hypothesis that the absence of natural light during winter induces a delay in chronotype and an increase in social jetlag. The influence of the photoperiod on sleep and circadian patterns has already been established^28^. Our findings concerning the delayed chronotypes are in line with those that showed more than a half-hour delay in mid-sleep during winter among people living within extreme photoperiods^20^ and during a winter Antarctic campaign^22^. Considering that chronotype has been demonstrated as a consistent and stable measure at the individual level over the course of months^12^, changes in this variable might be attributed to the extreme photoperiod conditions. In the absence of a natural light cue and a defined timetable to follow, the endogenous period, which exceeds 24 h, gives rise to a new phase of entrainment. Although the main variable for studying circadian rhythms is the mid-sleep during work-free days, alternative phase markers, such as sleep onset and offset, may similarly show delays reinforcing the shifted phase modulated by variations in zeitgebers^29–31^. Furthermore, we assume that on work-free days, when individuals experience fewer external constraints such as work schedules, their sleep patterns tend to align more closely with their inherent biological rhythms. This alignment offers a clearer insight into their innate circadian rhythm. Social jetlag describes the discrepancy between biological rhythms and social timing^4,32^. Previous studies have shown a clear correlation between later chronotypes and greater amounts of social jetlag^33,34^, which was also evidenced in Antarctica during a summer expedition^24^. Due to the lack of a natural light cue, and the subsequently delayed chronotype, the differences in mid-sleep between workdays and work-free days became more evident during the polar night. Indeed, these findings emphasize the importance of social cues in synchronizing the circadian clock^35^. Our data suggest that as the day length decreased, the days with a set routine helped maintain a more stable phase of entrainment. Conversely, during the work-free days the lack of clear social cues provokes a delay in this phase, providing compelling evidence for the pivotal role of a fixed timetable in preserving the alignment of our rhythms.

Seasonality also impacted Sleep duration on workdays, showing fewer hours of sleep during winter. This result reaffirmed the idea that although the participants maintained their social cues, the absence of a clear and strong zeitgeber may deteriorate circadian synchronization^36^. Interestingly, despite the well-known role of social cues in synchronizing the circadian system^37^, our findings revealed that even though the crew adhered to a fixed schedule the influence of light as a zeitgeber remained stronger in modulating this variable. We hypothesized that in the absence of these robust social cues, the lack of natural light may have had a more significant impact on this parameter. The lack of alternation between day and night probably led to a circadian phase change, delaying the time at which participants went to sleep. We support the idea that fixed morning schedules during workdays, in which the crew had to wake up early, provoked a reduction in the amount of sleep. Our findings are consistent with those obtained in our previous research carried out at the same Antarctic station in which the sleep–wake cycle was recorded by actigraphy^26^. These results also reinforced the fact that naps may play a key role in compensating for the sleep debt during workdays, as the seasonal variation was not noticeable in total sleep measures^38^.

### Model 2. Chronotype modulation

As expected, Model 2 evidenced that the chronotype modulates social jetlag. Previous studies have demonstrated this relationship in other contexts^39–41^. Our results are consistent with those showing that later chronotypes had greater social jetlag during a summer Antarctic campaign^24^. Based on our findings and in line with previous evidence, it appears that individuals with later chronotypes adjusted all their sleep phases to later hours in accordance with their endogenous rhythm^42^. Data indicated that these shifted phases were not limited to only work-free days, but were also evident on workdays when adhering to a work routine was necessary. However, the modulation of social jetlag by chronotype allows us to infer that, despite the association between chronotype and phase delay being observed on both workdays and work-free days, fixed routines still contribute to the synchronization of the circadian rhythm. Additionally, early chronotypes slept less during work-free days than the later ones. We speculate that later chronotypes had a greater sleep debt than the early chronotypes due to the delayed sleep onset and the work schedule. As a result, they needed to compensate for these hours during the weekend^43^.

## Limitations

In order to comprehensively examine the circadian variations resulting from the extreme photoperiod, it would have been valuable to include a control group for comparison. However, replicating the isolated and confined conditions, as well as the strict schedule carried out by military personnel in Antarctica throughout one year, presents considerable obstacles. Furthermore, addressing physiological measures like dim-light melatonin onset and sleep–wake cycle through actigraphy would have provided a complete overview of the processes under study allowing a better understanding of them. In addition, despite the crewmembers’ exposure to artificial light was mild^44^, measuring it could have yielded greater insights into its role as a zeitgeber. Lastly, increasing our size sample would have let us incorporate other variables as predictors in the models, enriching our research perspective.

## Conclusions

Our study provides evidence about the impact of the lack of natural light on chronotype and sleep measures, with the strength of being conducted through longitudinal research conducted in a naturalistic environment. These results shed light on the circadian misalignment caused by extreme photoperiod exposure and the role of strong social cues in a relatively large sample for this experimental setting. Though not directly applicable to other contexts, our results could be useful in the study of shift workers^45,46^, mineworkers^47^, medical staff^48^, and astronauts^49^. Future studies should explore how these changes can be associated with other variables that have already been shown to have a circadian modulation, such as alertness or physical and cognitive performance^50,51^.

## Materials and methods

### Participants and design

Belgrano II is an Argentine Antarctic station located approximately 1300 km from the South Pole (the 3rd southernmost permanent station of the planet), at sea level on the mainland at the Nunatak Bertrab (77° 51′ S and 34° 33′ W). Due to its southern position, Belgrano II is characterized by an extreme photoperiod consisting of four months of absence of natural light (polar night; from May to August), four months with constant daylight (polar day; from November to February), and four transition months between these two phases, with variable day lengths. The extreme isolation and confined conditions are also a singularity of this station. Each year, a crew of around 15 military personnel travel to Belgrano II. They usually arrive by February and remain for a year without any other possibility of contact rather than radio communications and internet. In the event of an emergency, a rescue team can take several days to reach Belgrano II from another Antarctic station, provided there are good weather conditions.

The crew has an established routine of five workdays from 9:00 am to 6:30 pm. On workdays, a reveille call signals the official start of the day at the station, while on days off, participants wake up at their own discretion. Each member has a designated task to perform, and two work-free days in which they have to develop only the main procedures to maintain the facilities. During the winter, the light–dark cycle depends on artificial lighting. Except for the medical room, all areas maintained illumination levels below 500 lx^26^.

Data were collected during five winter campaigns (2014, 2016–2019) and comprised pooled information from 82 participants who voluntarily agreed to join this observational, analytical and longitudinal study. The sample consisted of only men with a mean age of 35 ± 5 years and similar anthropometric characteristics (Body mass index [BMI] = 27 ± 3 kg/m^2^). Measures were assessed every other month from March to November. To rule out possible depression and anxiety disorders throughout the campaigns, that may influence the sleep variables^52^, the Beck Depression Inventory-II (BDI-II:^53^; Spanish version:^54^ and the Beck Anxiety Inventory (BAI:^55^; Spanish version:^56^ were assessed every other month throughout the year. Mood remained stable during the campaigns with scores of depression and anxiety that did not meet the criteria for mental disorder (DSM-IV; American Psychiatric Association, 1996). Non-significant variations were found between each measurement point (BAI *p* < 0.71; BDI-II *p* < 0.48) [data not shown].

The study was approved by the Ethics Committee from the National University of Quilmes (Bernal, Argentina) and performed in accordance with the Declaration of Helsinki and its amendments. Participants provided written informed consent after being informed about the nature and purpose of the study.

### Chronotype and sleep measures

The Munich Chronotype Questionnaire (MCTQ) is a self-report scale designed to assess chronotype. It inquiries about wake-up time, bedtime, sleep time, and naps during workdays and work-free days, assuming a structured work schedule^57^. The variables included in the analyses were: Mid-sleep on workdays (MSW); Mid-sleep on work-free days (MSF); Social jetlag which describes the discordance between an individual’s endogenous rhythm and the external social timing influenced by factors such as alarm clocks, work schedule, meals and social activities^58^ [MSF–MSW]; Sleep duration on workdays (SDW); Sleep duration on work-free days (SDF); Naps on workdays (NW); Naps on work-free days (NF); Total sleep on workdays [SDW + NW]; and Total sleep on work-free days [SDF + NF]; Sleep onset on workdays; Sleep onset on work-free days; Sleep offset on workdays; Sleep offset on work-free days; Sleep onset latency on workdays; Sleep onset latency on work-free days; Sleep inertia on workdays; Sleep inertia on work-free days; Time to bed on workdays; and Time to bed on work-free days. The following equation, which was utilized to obtain the chronotype’s value corrects the MSF for sleep debt on workdays (MSFsc)^58^:

$${\text{MSFsc}} \; = \;{\text{MSF}} {-} \frac{{\left[ {{\text{SDF}} - \frac{{\left( {5 \; \times \;{\text{SDW }} + 2 \; \times \;{\text{SDF}}} \right)}}{7}} \right]}}{2}$$

### Statistical analysis

Hierarchical linear modeling (HLM)^59^ was performed to examine seasonal chronotype variations and seasonal sleep parameters variations. We structured our data in two levels: the repeated measures level (Month), nested within the participant-level data.

**Figure 2:**
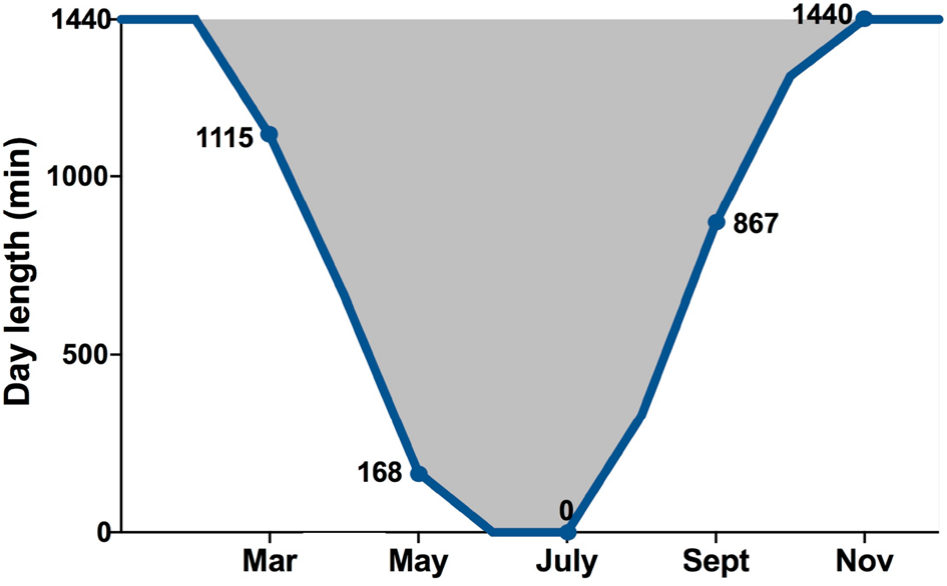
Day length at Belgrano II. Each point represents the average duration of natural sunlight throughout the year at Belgrano II Antarctic station. The curve illustrates the day length at each measurement point. Daylight is depicted by the color white, while the absence of natural light is shown in grey (https://www.timeanddate.com).

Two models were tested for each variable as obtained through the MCTQ. (1) The first model was performed to characterize the seasonal modulation of chronotype and sleep variables (Model 1. Seasonal modulation). Day length, measured as the differences between the end and start times of civil twilight^60^, was also included in the model. Day length was calculated averaging the total minutes of natural light during the first and the last day of each measurement point (https://www.timeanddate.com) (Fig. 2). (2) A conditional model was conducted including the chronotype at each measurement point, as a level 1 predictor of sleep variations (Model 2. Chronotype modulation). All analyses were performed with R Studio software while plots were constructed using Prism9 (GraphPad Software, La Jolla California, USA).

## Data Availability

The datasets generated and analysed during the current study are available from the corresponding author on reasonable request.

## References

[1] Takahashi JS, Menaker M (1982). Role of the suprachiasmatic nuclei in the circadian system of the house sparrow, Passer domesticus. J. Neurosci. Off. J. Soc. Neurosci..

[2] Roenneberg T, Merrow M (2016). The circadian clock and human health. Curr. Biol. CB.

[3] Roenneberg T, Pilz LK, Zerbini G, Winnebeck EC (2019). Chronotype and social jetlag: A (self-) critical review. Biology.

[4] Roenneberg T, Wirz-Justice A, Merrow M (2003). Life between clocks: Daily temporal patterns of human chronotypes. J. Biol. Rhythms.

[5] Horne JA, Ostberg O (1976). A self-assessment questionnaire to determine morningness-eveningness in human circadian rhythms. Int. J. Chronobiol..

[6] Reis C, Madeira SG, Lopes LV, Paiva T, Roenneberg T (2020). Validation of the Portuguese variant of the Munich chronotype questionnaire (MCTQPT). Front. Physiol..

[7] Farkova E, Novak JM, Mankova D, Koprivova J (2020). Comparison of Munich chronotype questionnaire (MCTQ) and morningness-eveningness questionnaire (MEQ) Czech version. Chronobiol. Int..

[8] Kantermann T, Sung H, Burgess HJ (2015). Comparing the morningness-eveningness questionnaire and Munich ChronoType questionnaire to the dim light melatonin onset. J. Biol. Rhythms.

[9] Santisteban JA, Brown TG, Gruber R (2018). Association between the Munich Chronotype questionnaire and wrist actigraphy. Sleep Disord..

[10] Ghotbi N (2020). The microMCTQ: An ultra-short version of the Munich ChronoType questionnaire. J. Biol. Rhythms.

[11] Roenneberg T (2007). Epidemiology of the human circadian clock. Sleep Med. Rev..

[12] Kantermann T, Eastman CI (2018). Circadian phase, circadian period and chronotype are reproducible over months. Chronobiol. Int..

[13] Miguel M, Oliveira VCD, Pereira D, Pedrazzoli M (2014). Detecting chronotype differences associated to latitude: A comparison between Horne–Östberg and Munich Chronotype questionnaires. Ann. Hum. Biol..

[14] Goldin AP, Sigman M, Braier G, Golombek DA, Leone MJ (2020). Interplay of chronotype and school timing predicts school performance. Nat. Hum. Behav..

[15] Ritonja J, Aronson KJ, Day AG, Korsiak J, Tranmer J (2018). Investigating cortisol production and pattern as mediators in the relationship between shift work and cardiometabolic risk. Can. J. Cardiol..

[16] Shawa N, Rae DE, Roden LC (2018). Impact of seasons on an individual's chronotype: Current perspectives. Nat. Sci. Sleep.

[17] Vollmer C, Randler C, Di Milia L (2012). Further evidence for the influence of photoperiod at birth on chronotype in a sample of German adolescents. Chronobiol. Int..

[18] Sladek M, Kudrnacova Roschova M, Adamkova V, Hamplova D, Sumova A (2020). Chronotype assessment via a large scale socio-demographic survey favours yearlong standard time over daylight saving time in central Europe. Sci. Rep..

[19] Leocadio-Miguel MA (2017). Latitudinal cline of chronotype. Sci. Rep..

[20] Friborg O, Rosenvinge JH, Wynn R, Gradisar M (2014). Sleep timing, chronotype, mood, and behavior at an Arctic latitude (69 degrees N). Sleep Med..

[21] Tortello C, Barbarito M, Cuiuli JM, Golombek DA, Vigo DE, Plano SA (2018). Psychological adaptation to extreme environments: Antarctica as a space analogue. Psychol. Behav. Sci. Int. J..

[22] Chen N (2016). Circadian rhythm and sleep during prolonged Antarctic residence at Chinese Zhongshan station. Wilderness Environ. Med..

[23] Premkumar M, Sable T, Dhanwal D, Dewan R (2013). Circadian levels of serum melatonin and cortisol in relation to changes in mood, sleep, and neurocognitive performance, spanning a year of residence in Antarctica. Neurosci. J..

[24] Tassino B, Horta S, Santana N, Levandovski R, Silva A (2016). Extreme late chronotypes and social jetlag challenged by Antarctic conditions in a population of university students from Uruguay. Sleep Sci..

[25] Silva A (2019). Chronotype-dependent changes in sleep habits associated with dim light melatonin onset in the Antarctic summer. Clocks Sleep.

[26] Folgueira A (2019). Sleep, napping and alertness during an overwintering mission at Belgrano II Argentine Antarctic station. Sci. Rep..

[27] Allebrandt KV (2014). Chronotype and sleep duration: the influence of season of assessment. Chronobiol. Int..

[28] Wright KP (2013). Entrainment of the human circadian clock to the natural light-dark cycle. Curr. Biol. CB.

[29] Kennaway DJ, Van Dorp CF (1991). Free-running rhythms of melatonin, cortisol, electrolytes, and sleep in humans in Antarctica. Am. J. Physiol. Regul. Integr. Comp. Physiol..

[30] Aschoff J, Wever R (1976). Human circadian rhythms: A multioscillatory system. Fed. Proc..

[31] de Blasiis K, Mauvieux B, Elsworth-Edelsten C, Pezé T, Jouffroy R, Hurdiel R (2019). Photoperiod impact on a sailor’s sleep-wake rhythm and core body temperature in polar environment. Wilderness Environ. Med..

[32] Wittmann M, Dinich J, Merrow M, Roenneberg T (2006). Social jetlag: misalignment of biological and social time. Chronobiol. Int..

[33] Arora T, Taheri S (2015). Associations among late chronotype, body mass index and dietary behaviors in young adolescents. Int. J. Obes. (Lond.).

[34] Takahashi M (2018). Chronotype and social jetlag influence human circadian clock gene expression. Sci. Rep..

[35] Mistlberger RE, Skene DJ (2004). Social influences on mammalian circadian rhythms: Animal and human studies. Biol. Rev. Camb. Philos. Soc..

[36] Sletten TL (2022). The role of circadian phase in sleep and performance during Antarctic winter expeditions. J. Pineal Res..

[37] Aschoff J (1971). Human circadian rhythms in continuous darkness: Entrainment by social cues. Science.

[38] Dhand R, Sohal H (2006). Good sleep, bad sleep! The role of daytime naps in healthy adults. Curr. Opin. Pulm. Med..

[39] Zhang Z, Cajochen C, Khatami R (2019). Social jetlag and chronotypes in the Chinese population: Analysis of data recorded by wearable devices. J. Med. Internet Res..

[40] Juda M, Vetter C, Roenneberg T (2013). Chronotype modulates sleep duration, sleep quality, and social jet lag in shift-workers. J. Biol. Rhythms.

[41] Levandovski R (2011). Depression scores associate with chronotype and social jetlag in a rural population. Chronobiol. Int..

[42] Reiter AM, Roach GD, Sargent C (2022). The night before night shift: Chronotype impacts total sleep and rapid eye movement sleep during a strategically delayed sleep. J. Sleep Res..

[43] Roepke SE, Duffy JF (2010). Differential impact of chronotype on weekday and weekend sleep timing and duration. Nat. Sci. Sleep.

[44] Cho Y, Ryu SH, Lee BR, Kim KH, Lee E, Choi J (2015). Effects of artificial light at night on human health: A literature review of observational and experimental studies applied to exposure assessment. Chronobiol. Int..

[45] Diez JJ (2020). Sleep misalignment and circadian rhythm impairment in long-haul bus drivers under a two-up operations system. Sleep Health.

[46] Boivin DB, Boudreau P, Kosmadopoulos A (2022). Disturbance of the circadian system in shift work and its health impact. J. Biol. Rhythms.

[47] Ferguson SA, Kennaway DJ, Baker A, Lamond N, Dawson D (2012). Sleep and circadian rhythms in mining operators: Limited evidence of adaptation to night shifts. Appl. Ergon..

[48] Rabstein S (2019). Differences in twenty-four-hour profiles of blue-light exposure between day and night shifts in female medical staff. Sci. Total Environ..

[49] Brainard GC, Barger LK, Soler RR, Hanifin JP (2016). The development of lighting countermeasures for sleep disruption and circadian misalignment during spaceflight. Curr. Opin. Pulm. Med..

[50] Lok R, Joyce DS, Zeitzer JM (2022). Impact of daytime spectral tuning on cognitive function. J. Photochem. Photobiol. B Biol..

[51] Valdez P (2019). Circadian rhythms in attention. Yale J. Biol. Med..

[52] Tortello C (2021). Coping with Antarctic demands: Psychological implications of isolation and confinement. Stress Health J. Int. Soc. Investig. Stress.

[53] Beck AT, Steer RA, Ball R, Ranieri W (1996). Comparison of Beck depression inventories -IA and -II in psychiatric outpatients. J. Pers. Assess..

[54] Brenlla, M. E. & Rodriguez, C. M. Adaptación argentina del inventario de depresión de Beck (BDI-II). *Paidos* 11–37 (2006).

[55] Beck AT, Epstein N, Brown G, Steer RA (1988). An inventory for measuring clinical anxiety: Psychometric properties. J. Consult. Clin. Psychol..

[56] Magan I, Sanz J, Garcia-Vera MP (2008). Psychometric properties of a Spanish version of the Beck anxiety inventory (BAI) in general population. Span. J. Psychol..

[57] Reiter AM, Sargent C, Roach GD (2021). Concordance of chronotype categorisations based on dim light melatonin onset, the morningness-eveningness questionnaire, and the Munich chronotype questionnaire. Clocks Sleep.

[58] Roenneberg T, Allebrandt KV, Merrow M, Vetter C (2012). Social jetlag and obesity. Curr. Biol. CB.

[59] Raudenbush SW, Bryk AS (2002). Hierarchical Linear Models: Applications and Data Analysis Methods.

[60] Brown TM (2020). Melanopic illuminance defines the magnitude of human circadian light responses under a wide range of conditions. J. Pineal Res..

